# High-order spectral/*hp* element discretisation for reaction–diffusion problems on surfaces: Application to cardiac electrophysiology^☆^

**DOI:** 10.1016/j.jcp.2013.10.019

**Published:** 2014-01-15

**Authors:** Chris D. Cantwell, Sergey Yakovlev, Robert M. Kirby, Nicholas S. Peters, Spencer J. Sherwin

**Affiliations:** a National Heart and Lung Institute, Imperial College London, London, UK; b School of Computing and Scientific Computing and Imaging (SCI) Institute, Univ. of Utah, Salt Lake City, UT, USA; c Department of Aeronautics, Imperial College London, London, UK

**Keywords:** High-order finite elements, Spectral/*hp* elements, Continuous Galerkin method, Surface PDE, Cardiac electrophysiology, Monodomain equation

## Abstract

We present a numerical discretisation of an embedded two-dimensional manifold using high-order continuous Galerkin spectral/*hp* elements, which provide exponential convergence of the solution with increasing polynomial order, while retaining geometric flexibility in the representation of the domain. Our work is motivated by applications in cardiac electrophysiology where sharp gradients in the solution benefit from the high-order discretisation, while the computational cost of anatomically-realistic models can be significantly reduced through the surface representation and use of high-order methods. We describe and validate our discretisation and provide a demonstration of its application to modelling electrochemical propagation across a human left atrium.

## 1 Introduction

Partial Differential Equations (PDEs) describe many physical and mathematical processes and are quite often posed on embedded surfaces. PDEs on arbitrary surfaces rarely have analytic solutions and so are solved numerically using, for example, finite difference or finite element techniques. In the case of physical processes, a surface representation of the domain is usually an approximation of the true system and is made for numerical efficiency reasons providing it does not overly degrade the underlying physics. There are many examples of applications where PDEs are solved on surfaces in the literature, including fluid dynamics [1], biology and medicine [2], and computer graphics [3]. In this paper we describe a formulation of high-order spectral/*hp* element methods on curvilinear codimension-one surfaces embedded in three-dimensional space, applied to modelling electrical propagation in the heart.

The standard approach to solving a PDE on an embedded domain using finite element methods is to discretise the surface using a triangulation [4–6], or to parametrise the surface [1]. The latter may be challenging for arbitrary surfaces or require the use of multiple patches. Triangulation may lead to discretisation errors due to poor representation of the surface. It may also be computationally expensive in cases where the surface evolves in time. Finite volume methods have also been considered (for example, [7]), but are not examined further here. Alternative approaches include the level-set [8] or closest point method [9,10]. Level-set methods describe the surface as the zero level-set of a, possibly time-dependent, function ϕ(x,t) and extend the PDE from this surface into a higher-dimensional ambient Euclidean space. The extended PDE is solved using finite difference [11,12] or finite element [3,13] techniques. This removes much of the complexity of constructing operators on the manifold, but at the expense of increasing the dimension of the problem and, therefore, the computational cost. Care must also be taken to ensure the extended PDE remains true to the original surface PDE and complications can arise due to the necessity to impose boundary conditions on either side of the surface which may lead to an artificial jump in the solution, degeneracy of the implicit equation and difficulties in ensuring regularity of the solutions [14], although some of these can be addressed [15]. However, these methods allow the surface to move in time, in a relatively computationally efficient manner.

Mathematical formulations for solving PDEs on surfaces using linear finite element methods have been considered previously for a number of applications. One of the most prominent in the literature is the solution of the shallow water equations on the Earthʼs surface, for example [16], where the local coordinate systems on each element eliminate the singularities inherent when solving in global spherical coordinates. Spectral elements have also been used for the shallow water equations on a sphere by Giraldo [17] and Taylor et al. [18]. They find, for realistic atmospheric problems that these methods achieve comparable accuracy to existing methods, although they anticipate the methods can be more powerful when using local refinement. Their study is also restricted to a parametrised sphere using quadrilaterals. Finally, PDEs on surfaces are important in computer graphics [3] for rendering and texturing on surfaces and visualising flow data from simulations [19]. Fluid flow simulations on surfaces of arbitrary topology have been discussed by Stam [1].

In this paper we are interested in defining spectral/*hp* element discretisations of surfaces of arbitrary and complex geometry as are typically found in biomedical applications. Such surfaces are frequently extracted from medical imaging: a global surface parametrisation is infeasible. Instead we tessellate the surface with geometrically high-order curvilinear elements, each defined by a mapping from a planar reference region. The mapping is then incorporated into the differential operators during their construction. The particular application we consider is the simulation of human left atrium electrophysiology. We discard the mechanical aspects of the heart, so do not require the capabilities to model moving surfaces.

### 1.1 Cardiac electrophysiology

Cardiac conduction occurs due to a complex sequence of ion transport mechanisms between cells. As ions flow from adjacent excited cells and the potential difference across the cell membrane exceeds a threshold level, a complex sequence of ionic currents begins to flow between the intracellular and extracellular spaces creating a prescribed variation of the transmembrane potential known as the action potential. This process begins with a complete and rapid depolarisation of the cell due to the inward sodium current. Other ionic currents gradually restore the polarised state to complete the cycle. The depolarisation of the cell causes contraction and the cumulative effect results in coordinated contraction of the heart muscle with each activation wave. In some cases, due to disease, infarction or age, inhomogeneities in the myocardium result in abnormal activation patterns, known as cardiac arrhythmias, leading to irregular contraction of the heart and poor cardiac throughput. If such dysfunction occurs in the ventricles it is rapidly fatal, causing cessation of effective blood circulation, and is the most common cause of cardiac arrest. When occurring in the atria, it causes symptoms such as tiredness and leaves the person prone to the formation of blood clots in the poorly contracting atrium and puts them at greater risk of stroke.

Identifying those areas of myocardium responsible for the initiation or perpetuation of an arrhythmia is key to successful clinical intervention and therefore accurate and rapid computer simulation of a patientʼs atrial electrical activity is potentially a highly valuable tool in planning treatment. Significantly improving the performance of cardiac electrophysiology computer simulations is key for them to attain clinical utility. Spectral elements are capable of providing high-resolution solutions, necessary to capture the sharp gradients at the leading edge of the depolarisation wave, with fewer degrees of freedom than linear finite element methods [20]. In addition, these high-order methods can be extended to perform local polynomial refinement where needed during the simulation, such as on those elements in close proximity to the wavefront, without needing to resort to computationally expensive mesh refinement.

**Fig. 1:**
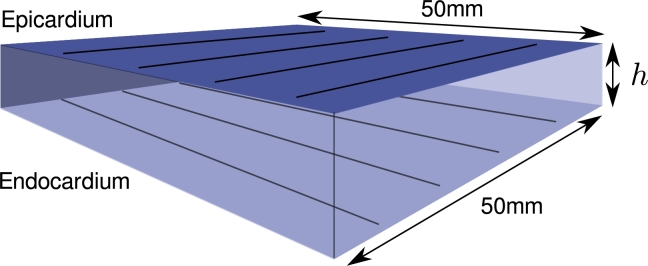
Diagram of the 25 cm^2^ slice geometry of thickness *h* mm used to quantify three-dimensional effects which cannot be captured by a surface approximation. Conductivities for the anisotropic case reflect orthogonal fibre directions on the epicardial and endocardial surfaces with continuous gradation inbetween. For the isotropic case, conductivities are thickness-averaged. Stimulus is initiated from the point (0,0,*h*/2), located at the centre of the slice.

**Fig. 2:**
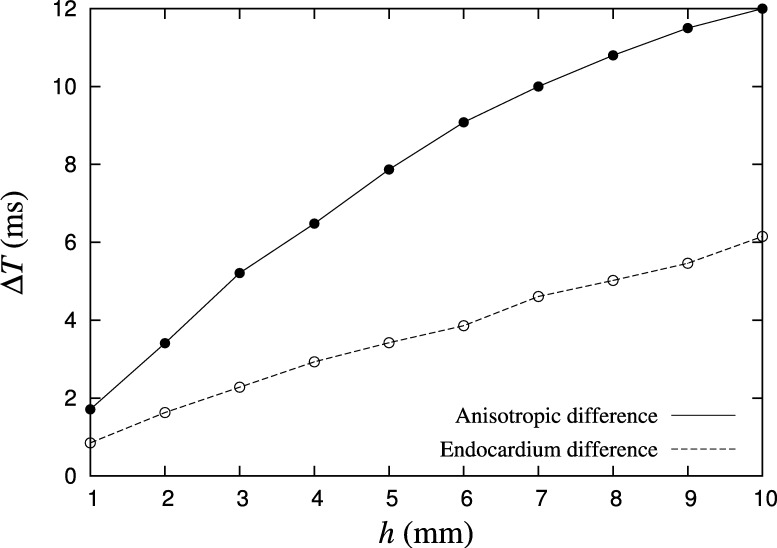
Maximum difference Δ*T* in local activation time between the epicardial and endocardial surfaces (anisotropic difference), and between the endocardial surfaces of the anisotropic and isotropic cases (endocardium difference).

Simplifying the geometric model of the atrium is another approach to accelerating simulations. To date most computer simulations of mammalian atria represent the chamber walls as fully three-dimensional substrate, typically from a volume segmentation of magnetic resonance angiography (MRA) images. The wall of the human atrium is typically only 1–3 mm thick, but with a surface area in excess of 50 cm^2^. Electrical propagation and arrhythmogenic features are therefore predominantly two-dimensional in nature and can be efficiently modelled as a two-dimensional surface. To illustrate this, we quantify the effect of transmural variability in a 3D tissue slice, described in Fig. 1, when the epicardial and endocardial surfaces have orthogonal fibre directions. Additionally, we compare this to the same geometry using isotropic conductivities, computed as the transmural average of those in the anisotropic case. In each case, the tissue is stimulated from the centre point of the tissue with a current of 50 μA, applied for 2 ms over a spherical region of radius 3 mm. Fig. 2 shows the maximum difference in activation time between the epicardial and endocardial layers for the anisotropic case, as well as a comparison between the endocardial layers of the isotropic and anisotropic cases. For a thickness of 2 mm – the average thickness of the left atrial wall – the maximum difference in local activation time between corresponding points on the epicardial and endocardial surface is 3.41 ms. In comparing the endocardial surfaces of the anisotropic and isotropic cases, which is most relevant for clinical applications where electrical recordings are taken endocardially, the maximum difference is 1.63 ms. This variation is less than 0.33% of the human atrial action potential, which is in excess of 300 ms in healthy myocardium. Representing the left atrial geometry as a two-dimensional manifold can therefore substantially reduce the computational cost of simulations when compared to full three-dimensional tissue models, without a clinically significant loss of precision.

The paper is structured as follows. In Section 2 we outline the mathematical construction of the manifold embedding along with the high-order discretisation technique used. Section 3 describes a number of test problems with analytic solutions used to verify the formulation and implementation. We conclude this section with an applied demonstration of the technique using a model of cardiac electrophysiology in the human left atrium. We summarise and discuss implementational details in Section 5.

## 2 Formulation

A mathematical formulation of the surface embedding within $R^{3}$ along with the corresponding numerical construction of the continuous Galerkin approximation on the surface and subsequent implementation now follows. In this section we limit ourselves to an outline of the mathematical derivation of the Laplace–Beltrami operator on the manifold, and include a more rigorous derivation in Appendix A.

In constructing operators on a curved surface one must take care to distinguish between vector quantities which are geometrically fixed and independent of the coordinate system in which they are represented, and those which are inherently coupled to the chosen coordinate system. The former is known as *contravariance*; a contravariant vector, such as velocity, is one in which under a change of basis the components must change under the inverse map to retain geometric invariance. In contrast, the latter is termed *covariance* and such vector quantities, for example the gradient of a scalar function, change with the basis transformation. More details on these concepts and their relationship can be found in [21]. In Euclidean space there is no distinction between these two concepts; however, this is not true in curvilinear embedded spaces. We first define our manifold through a coordinate mapping and outline the geometrical properties of this mapping. This allows generic representations of familiar differential operators on the manifold surface to be constructed.

### 2.1 Notation

Our formulation of differential operators on the surface is derived in terms of first- and second-order tensors. Accordingly, we take advantage of the notational conventions from [21]. In brief, a tensor is denoted in bold (e.g. ***a***), while entries within a tensor are denoted with indices as necessitated by the order of the tensor (e.g. $a_{ij}$ for a second-order tensor). Furthermore, lower indices refer to covariant quantities while upper indices denote contravariant quantities. Tensors may themselves be indexed and this is denoted through the use of indices enclosed in parentheses (e.g. $a_{\left(i\right)}$). Vector quantities are denoted in bold, since they are tensors of one dimension. Unless specifically stated otherwise, all differential operators in this section are restricted to the surface.

### 2.2 Metric tensors and differential operators

For a smooth codimension-1 manifold $M\subset R^{3}$ we express coordinate directions on the manifold using $\xi^{i}$ and in the ambient space using $x^{i}$. The surface is parametrised using the coordinate mapping $x:M\to R^{3}$ for which the entries of the second-order Jacobian tensor are given by

$$J_{i}^{j}=\frac{\partial x^{j}}{\partial \xi^{i}},$$

where $J_{i}^{j}$ can be viewed as a covariant surface vector (by fixing the upper index) or as a contravariant space vector (by fixing the lower index). At each point *p* on the surface *M*, the tangent plane $T_{p}M$ is a real vector space spanned by the vectors,

(1) $$t_{\left(i\right)}=\left(\frac{\partial x^{1}}{\partial \xi^{i}},\frac{\partial x^{2}}{\partial \xi^{i}},\frac{\partial x^{3}}{\partial \xi^{i}}\right),\;i=1,2.$$

The surface metric tensor provides important information about how lengths and angles vary on the manifold surface. This second-order tensor is constructed as the pairwise inner product of tangent vectors, $g_{ij}=t_{\left(i\right)}\cdot t_{\left(j\right)}$, and has determinant g=|g|. The tensor ***g*** can be compactly expressed in terms of the Jacobian tensor of ***x*** as $g={JJ}^{⊤}$. Note that ***g*** is square and the determinant is well-defined. The Jacobian tensor is not square and therefore the conventional Jacobian determinant, frequently used in finite element codes, is undefined. The metric ***g*** is used when mapping contravariant quantities to covariant quantities. To obtain contravariant vectors, which must remain fixed under a change of coordinate system, from covariant vectors we will require the inverse of the metric tensor, $g^{-1}$ for which we denote entries as $g^{ij}$, in order to transform the components of these vectors accordingly.

In the curved space of the manifold the derivative of a scalar quantity, *f*, remains consistent with that of the conventional Euclidean definition,

(2) $$\nabla_{k}f=\frac{\partial f}{\partial x^{k}},$$

since it is a covariant quantity and changes with the coordinate system. However when taking derivatives of vector quantities, **v**, the curvature of the space must be taken into account when calculating the change in the vector components. It can be appreciated that Euclidean derivatives of a covariant vector at a point *p* on a manifold *M* do not necessarily lie in the tangent plane to the manifold, $T_{p}M$. Therefore we must define the covariant derivative operator in such a way that the result remains on the manifold and accounts for the curvature of the surface. The covariant derivative of a contravariant vector ***v*** is therefore

$$\nabla_{k}v^{i}=\frac{\partial v^{i}}{\partial x^{k}}+\sum_{j=1}^{2}v^{j}\Gamma_{jk}^{i},$$

where $\Gamma_{jk}^{i}$ are the *Christoffel symbols* [21]. These real-valued quantities capture the change in the tangent vectors $t_{\left(i\right)}$ as *p* moves on the manifold. By expressing the Christoffel symbols in terms of the derivatives of the metric tensor entries $g_{ij}$ (see Appendix A), we can express the surface divergence operator as

(3) $$\nabla \cdot v=\sum_{k=1}^{2}\nabla_{k}v^{k}=\frac{1}{\sqrt{g}}\sum_{k=1}^{2}\frac{\partial (v^{k}\sqrt{g}\;)}{\partial \xi^{k}}.$$

Using $\nabla^{i}f=\sum_{j=1}^{2}g^{ij}\nabla_{j}f$ to transform the covariant scalar gradient vector to a corresponding contravariant vector and combining this with the above expression for divergence, the Laplacian operator on the manifold can be expressed as

(4) $$\Delta_{M}f=\sum_{i=1}^{2}\nabla_{i}\nabla^{i}f=\frac{1}{\sqrt{g}}\sum_{i=1}^{2}\frac{\partial (\sqrt{g}\;\nabla^{i}f)}{\partial \xi^{i}}=\frac{1}{\sqrt{g}}\sum_{i=1}^{2}\sum_{j=1}^{2}\frac{\partial }{\partial \xi^{i}}\left(\sqrt{g}\;g^{ij}\frac{\partial f}{\partial \xi^{j}}\right).$$

This can be extended to the anisotropic case, as derived in Appendix A.3.

### 2.3 Spectral/*hp* element discretisation

A spatial discretisation of the manifold surface is given using the spectral/*hp* element method. A more detailed description of the basis construction, given in the context of fluid dynamics, can be found in [22]. The computational domain Ω=M is a non-overlapping tessellation of elemental regions $\Omega^{e}$ such that $\Omega ={⋃}_{e}\Omega_{e}$ and the intersection of any two elements is either a point, an edge or the empty set. Standard elemental regions $\Omega_{st}(\xi_{1},\xi_{2})$ are defined for the triangular and quadrilateral regions, defined respectively as,

$$Q^{2}(\xi )=\left\{(\xi_{1},\xi_{2})\in {\left[-1,1\right]}^{2}\right\},$$

$$T^{2}(\xi )=\left\{(\xi_{1},\xi_{2}):-1⩽\xi_{i},\;i=1,2;\;\xi_{1}+\xi_{2}⩽0\right\},$$

and these are mapped to each $\Omega^{e}$ through smooth mappings $x_{e}(\xi^{1},\xi^{2})=(x_{e}^{1}(\xi^{1},\xi^{2}),x_{e}^{2}(\xi^{1},\xi^{2}),x_{e}^{3}(\xi^{1},\xi^{2}))$. In the case of the triangular region, we employ a coordinate transform [23,24] which allows it to be represented using the same fixed coordinate limits as used in the quadrilateral region,

$$T^{2}(\eta )=\left\{(\eta_{1},\eta_{2})\in {\left[-1,1\right]}^{2}\right\}.$$

On these reference regions $\Omega_{st}$ we represent a smooth function $u(\xi_{1},\xi_{2})$ in terms of a set of *N* basis functions, $\left\{\phi_{n}(\xi_{1},\xi_{2})\right\}$. The $\phi_{n}:{\left[-1,1\right]}^{2}\to R$ are constructed through a tensor product of two sets of $P_{1}+1$ and $P_{2}+1$ one-dimensional basis functions $\left\{\psi_{p}(\xi )\right\}$, with $\psi_{p}:[-1,1]\to R$, where we denote by $P_{i}$ the largest order of polynomial in the *i*-th basis, and $N=(P_{1}+1)(P_{2}+1)$. Typically, $P_{1}=P_{2}$ in most applications, but the sizes of the bases could be chosen differently. Typically, one chooses a subset of the family of Jacobi polynomials $P_{p}^{a,b}$ for basis functions, due to their inherent orthogonality properties and the resulting amenable mass matrix structure. The modal nature of these polynomials is more computationally amenable to *p*-refinement than the original nodal spectral element method since the stiffness matrices do not need to be entirely rebuilt, although we do not consider *p*-adaptivity here. In this paper, we specifically choose the Jacobi polynomials, $P_{p}^{1,1}$, but modify them with linear functions as,

$$\psi_{p}(\xi )=\left\{ \begin{array}{cc} \frac{1-\xi }{2} & \text{if }p=0,\\ \frac{1-\xi }{2}\frac{1+\xi }{2}P_{p-1}^{1,1}(\xi ) & \text{if }0<p<P,\\ \frac{1+\xi }{2} & \text{if }p=P \end{array}\right.$$

which naturally partitions the modes into element-interior modes and element-boundary modes, the latter of which has support which includes one or more edges of the element. An infinite-dimensional function *u* can therefore be projected into the polynomial space spanned by $\phi_{n}$ to give a discrete approximation $u^{\delta }$ on the element as

$$u^{\delta }(\xi_{1},\xi_{2})=\sum_{n=0}^{N-1}\phi_{n}(\xi_{1},\xi_{2}){\hat{u}}_{n}=\sum_{p=0}^{P_{1}}\sum_{q=0}^{P_{2}}\psi_{p}(\xi_{1})\psi_{q}(\xi_{2}){\hat{u}}_{pq},$$

where the ${\hat{u}}_{n}$ denote the degree of freedom quantifying the contribution of the basis function $\phi_{n}$.

In constructing a continuous Galerkin formulation, $C^{0}$-continuity is enforced across elemental boundaries. Corresponding boundary modes from adjacent elements form a single global mode in a domain-wide expansion. Since the support of the element-interior basis functions does not extend to, or beyond, the boundary of the element, they are by definition global modes in themselves. Mathematically, this assembly of element modes is expressed through an assembly matrix ***A***.

We build on this formulation by casting the Helmholtz equation into the weak Galerkin approximation on the manifold *M*,

(5) $$H(u)=\nabla \cdot \tilde{\sigma }\nabla u-\lambda u=f,\;\lambda >0,$$

where $\tilde{\sigma }$ is a surface diffusion tensor (see Appendix A.3) and *f* is a prescribed forcing function. Given the discrete approximation space

$$U=\left\{u\in {\left(L^{2}(\Omega )\right)}^{2}:u{|}_{\Omega_{e}}\in {\left(P^{p}(\Omega_{e})\right)}^{2},\;\forall \Omega_{e}\in \Omega \right\}$$

and denoting element and boundary integration by

$$(u,v)=\int_{\Omega }uv\;d\Omega \;\text{and}\;\langle u,v\rangle =\int_{\partial \Omega }uv\;ds$$

respectively, we choose the test space V=U and seek solutions u∈U such that (v,H(u))=(v,f), ∀v∈V. After integration by parts this gives the anisotropic variational form of Eq. (5) as

(6) $$-\sum_{k}\left(\sum_{i,j}{\tilde{\sigma }}_{j}^{k}g^{ij}\frac{\partial u}{\partial \xi^{i}},\frac{\partial v}{\partial \xi^{k}}\right)-\lambda (v,u)+\sum_{k}\langle \sum_{i,j}{\tilde{\sigma }}_{j}^{k}g^{ij}\frac{\partial u}{\partial \xi^{i}},n_{k}v\rangle =(v,f).$$

### 2.4 Implementation

The spectral/*hp* element method outlined in the previous section is implemented in the Nektar++ spectral/*hp* element framework [25]. In the weak form from Eq. (6) the function *u* is expanded in terms of the elemental basis functions $\phi_{n}$, to form an elementally discrete system which, in matrix form, can be expressed as

$$L^{e}{\hat{u}}^{e}+\lambda M^{e}{\hat{u}}^{e}-b^{e}{\hat{u}}^{e}=-{\hat{f}}^{e}$$

where

$$L^{e}[m][n]=\sum_{k}\int_{\Omega^{e}}\sum_{i,j}{\tilde{\sigma }}_{j}^{k}g^{ij}\frac{\partial \phi_{m}}{\partial \xi^{i}}\frac{\partial \phi_{n}}{\partial \xi^{k}}\;d\Omega^{e},$$

$$M^{e}[m][n]=\int_{\Omega^{e}}\phi_{m}\phi_{n}\;d\Omega^{e},$$

$$b^{e}[m][n]=\sum_{k}\int_{\partial \Omega^{e}}\sum_{i,j}{\tilde{\sigma }}_{j}^{k}g^{ij}\frac{\partial \phi_{m}}{\partial \xi^{i}}(n_{k}\cdot \phi_{n})\;ds,$$

$${\hat{f}}^{e}[m]=\int_{\Omega^{e}}\phi_{m}f\;d\Omega^{e}.$$

Elemental block matrices and concatenated vectors of element coefficients are assembled using a highly sparse global assembly matrix ***A***, which practically is implemented as an injective map for memory efficiency reasons, to create an expansion in terms of the global modes $\Phi_{n}$. The resulting global system of linear equations,

$$(L+\lambda M-b)\hat{u}=-\hat{f},$$

is solved for the coefficients $\hat{u}$ using a preconditioned conjugate gradient method. For high-order discretisations, the global matrix is statically condensed and first solved for the wireframe mesh (vertex and edge degrees of freedom). The performance of this step is predominantly dictated by the cost of the matrix–vector multiplication operation. At higher polynomial orders it is more efficient to perform the matrix–vector operation locally on boundary modes of each element and assemble the global result, rather than performing a sparse matrix–vector multiplication with the assembled matrix [26]. The interior matrix block of the statically condensed system, corresponding to the element-interior modes, is block diagonal and so can be trivially inverted to complete the solve.

In parallel, the mesh is partitioned across the available processes and those global degrees of freedom which lie on the partition boundaries are duplicated on the neighbouring processes. The assembly process then requires the exchange of elemental contributions using the Message Passing Interface (MPI) between each of the processes sharing partition-boundary modes. Practically, we use the gather–scatter algorithm from Nek5000 which has been shown to scale well up to many thousands of processors [27].

For unsteady problems the equations can be time-marched using one of a number of implicit or implicit–explicit (IMEX) time integration schemes [28], implemented using general linear methods [29]. In summary, the PDE is arranged in the form

$$\frac{\partial u}{\partial t}=f(u)+g(u),$$

where f(u) is typically nonlinear and therefore should be evaluated explicitly, and g(u) is stiff and therefore best evaluated implicitly so as to avoid excessively small time-steps. The first-order forward-/backward-Euler IMEX scheme given by

$$\frac{u^{n+1}-u^{n}}{\Delta t}=g\left(u^{n}\right)+f\left(u^{n+1}\right)$$

is used for the electrophysiology results in this paper.

### 2.5 Comparison with traditional finite element formulations

In the formulation of finite element methods in two-dimensional Euclidean space one requires a set of geometric terms in order to apply differential operators constructed on the standard reference region to the physical space element. These terms correspond directly to the inverse of the Jacobian of the mapping,

$${\left(J^{-1}\right)}_{j}^{i}=\frac{\partial \xi^{i}}{\partial x^{j}}.$$

In Euclidean spaces, the Jacobian is square and the inverse is well-defined. However, in the case of a higher-dimensional embedding, the Jacobian is rectangular and the inverse is not well-defined. Therefore, one approach to support existing finite element codes on a manifold is to extend the two-dimensional surface mapping to a full three-dimensional representation by artificially adding a third coordinate direction corresponding to the surface normal. This can be found through the cross product of the tangent vectors,

$$h=\left[\begin{array}{c} h_{1}\\ h_{2}\\ h_{3} \end{array}\right]=\frac{\partial x}{\partial \xi_{1}}\times \frac{\partial x}{\partial \xi_{2}}=\left[\begin{array}{c} \frac{\partial x_{2}}{\partial \xi_{1}}\frac{\partial x_{3}}{\partial \xi_{2}}-\frac{\partial x_{3}}{\partial \xi_{1}}\frac{\partial x_{2}}{\partial \xi_{2}}\\ \frac{\partial x_{3}}{\partial \xi_{1}}\frac{\partial x_{1}}{\partial \xi_{2}}-\frac{\partial x_{1}}{\partial \xi_{1}}\frac{\partial x_{3}}{\partial \xi_{2}}\\ \frac{\partial x_{1}}{\partial \xi_{1}}\frac{\partial x_{2}}{\partial \xi_{2}}-\frac{\partial x_{2}}{\partial \xi_{1}}\frac{\partial x_{1}}{\partial \xi_{2}} \end{array}\right].$$

A full 3×3 Jacobian matrix can be constructed by extending the 2×3 surface Jacobian with these additional terms,

$$\frac{\partial x_{1}}{\partial \xi_{3}}=h_{1},\;\frac{\partial x_{2}}{\partial \xi_{3}}=h_{2},\;\frac{\partial x_{3}}{\partial \xi_{3}}=h_{3}.$$

This matrix can be inverted to compute the values of $\frac{\partial \xi_{i}}{\partial x_{j}}$ as

$$\frac{\partial \xi_{1}}{\partial x_{1}}=\frac{1}{J_{3D}}\left(\frac{\partial x_{2}}{\partial \xi_{2}}h_{3}-\frac{\partial x_{3}}{\partial \xi_{2}}h_{2}\right),\;\frac{\partial \xi_{1}}{\partial x_{2}}=-\frac{1}{J_{3D}}\left(\frac{\partial x_{1}}{\partial \xi_{2}}h_{3}-\frac{\partial x_{3}}{\partial \xi_{2}}h_{1}\right),$$

$$\frac{\partial \xi_{1}}{\partial x_{3}}=\frac{1}{J_{3D}}\left(\frac{\partial x_{1}}{\partial \xi_{2}}h_{2}-\frac{\partial x_{2}}{\partial \xi_{2}}h_{1}\right),\;\frac{\partial \xi_{2}}{\partial x_{1}}=-\frac{1}{J_{3D}}\left(\frac{\partial x_{2}}{\partial \xi_{1}}h_{3}-\frac{\partial x_{3}}{\partial \xi_{1}}h_{2}\right),$$

$$\frac{\partial \xi_{2}}{\partial x_{2}}=\frac{1}{J_{3D}}\left(\frac{\partial x_{1}}{\partial \xi_{1}}h_{3}-\frac{\partial x_{3}}{\partial \xi_{1}}h_{1}\right),\;\frac{\partial \xi_{2}}{\partial x_{3}}=-\frac{1}{J_{3D}}\left(\frac{\partial x_{1}}{\partial \xi_{1}}h_{2}-\frac{\partial x_{2}}{\partial \xi_{1}}h_{1}\right),$$

where the full three-dimensional Jacobian determinant is

$$J_{3D}=h_{3}\left(\frac{\partial x_{1}}{\partial \xi_{1}}\frac{\partial x_{2}}{\partial \xi_{2}}-\frac{\partial x_{1}}{\partial \xi_{2}}\frac{\partial x_{2}}{\partial \xi_{1}}\right)+h_{2}\left(\frac{\partial x_{1}}{\partial \xi_{2}}\frac{\partial x_{3}}{\partial \xi_{1}}-\frac{\partial x_{1}}{\partial \xi_{1}}\frac{\partial x_{3}}{\partial \xi_{2}}\right)+h_{1}\left(\frac{\partial x_{2}}{\partial \xi_{1}}\frac{\partial x_{3}}{\partial \xi_{2}}-\frac{\partial x_{2}}{\partial \xi_{2}}\frac{\partial x_{3}}{\partial \xi_{1}}\right).$$

Finally, the surface Jacobian is computed as

$$J=\left|\frac{\partial x}{\partial \xi_{1}}\times \frac{\partial x}{\partial \xi_{2}}\right|=\sqrt{J_{3D}}.$$

The resulting terms of the divergence operator

$$\nabla \cdot v_{i}e_{i}=\sum_{j=1}^{2}\frac{\partial \xi_{j}}{\partial x}\frac{\partial v_{i}}{\partial \xi_{j}}$$

are mathematically equivalent to those obtained through the geometric tensor construction.

## 3 Validation

We present a number of simulations using the implementation in Nektar++ to validate the methodology and to demonstrate its capability to represent complex problems in the application area of cardiac electrophysiology. Problems with analytic solutions are first considered to verify the correct numerical properties are being observed. An applied example then follows in which we simulate the electrical activation of cells in the human left atrium, represented as a two-dimensional surface, using the monodomain reaction–diffusion equation.

For analytic test cases, we consider the computational domain to be the unit-radius sphere $S^{2}\subset R^{3}$, or a subset thereof. This domain is sufficiently complex to demonstrate the effectiveness of the method without degenerating to a relatively trivial problem, yet simple enough to retain mathematically elegant solutions for particular choices of parameters. The sphere is parametrised by the mapping from spherical to Cartesian coordinates,

χ(θ,ϕ)=(sinθcosϕ,sinθsinϕ,cosθ)

which results in the Jacobian, metric tensor, and inverse metric tensor,

$$J=\left(\begin{array}{ccc} \cos\theta \cos\phi & \cos\theta \sin\phi & -\sin\theta \\ -\sin\theta \sin\phi & \sin\theta \cos\phi & 0 \end{array}\right),\;g=\left(\begin{array}{cc} 1 & 0\\ 0 & \sin^{2}\theta \end{array}\right),\;g^{-1}=\left(\begin{array}{cc} 1 & 0\\ 0 & \frac{1}{\sin^{2}\theta } \end{array}\right),$$

respectively. Substitution into Eq. (4) gives the expression for the Laplacian operator on the spherical surface as

(7) $$\Delta_{M}u=\frac{1}{\sin\theta }\frac{\partial }{\partial \theta }\left(\sin\theta \frac{\partial u}{\partial \theta }\right)+\frac{1}{\sin\theta }\frac{\partial }{\partial \phi }\left(\frac{1}{\sin\theta }\frac{\partial u}{\partial \phi }\right)=\frac{\cos\theta }{\sin\theta }\frac{\partial u}{\partial \theta }+\frac{\partial^{2}u}{\partial \theta^{2}}+\frac{1}{\sin^{2}\theta }\frac{\partial^{2}u}{\partial \phi^{2}}.$$

In spherical coordinates, solutions to the Laplace equation can be obtained analytically through expansion in spherical harmonics,

$$Y_{\ell }^{m}(\theta ,\varphi )=C_{l,m}P_{\ell }^{m}(\cos\theta )e^{im\varphi },$$

where $P_{\ell }^{m}$ are the *associated Legendre polynomials* and $C_{l,m}$ are constants. Consequently, these functions are good candidates for testing the formulation of the Laplacian on a sphere. It is apparent that if we take m=0 to maintain real solutions and discard the constants for clarity we are interested in solutions of the form $Y_{\ell }^{0}(\theta ,\varphi )=P_{\ell }(\cos\;\theta )$, where $P_{\ell }(z)$ are the Legendre polynomials satisfying,

$$\frac{d}{dz}\left[\left(1-z^{2}\right)\frac{d}{dz}P_{n}(z)\right]+n(n+1)P_{n}(z)=0.$$

**Fig. 3:**
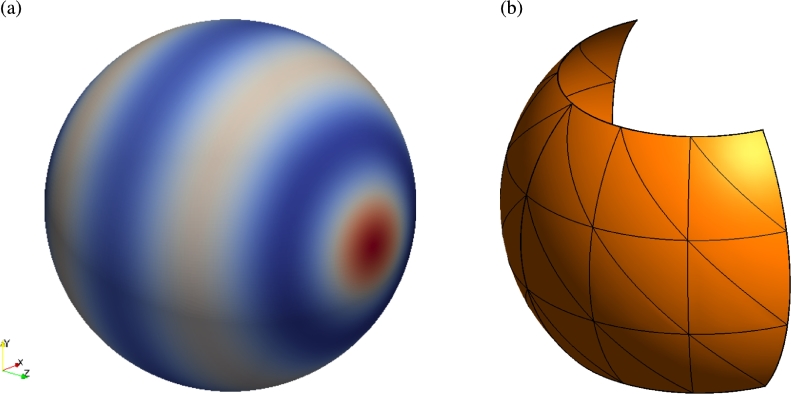
(a) Representative solution $u=P_{6}(\cos\;\theta )$. Such functions are spherical harmonics which are analytic solutions to the Laplace equation in spherical coordinates. (b) High-order mesh of a patch of the unit sphere given by $\Omega =\{S^{2}(r,\theta ,\phi )|(\theta ,\phi )\in [-\frac{\pi }{4},\frac{\pi }{4}]\times [-\frac{\pi }{2},\frac{\pi }{2}],\;r=1\}$. This example consists of 36 triangular elements. The equivalent quadrilateral mesh consists of 18 elements obtained through recombining pairs of triangles.

Choosing z=cosθ, and applying the chain rule,

$$\frac{\cos\theta }{\sin\theta }\frac{d}{d\theta }\left(P_{n}(\cos\theta )\right)+\frac{d^{2}}{d\theta^{2}}\left(P_{n}(\cos\theta )\right)+n(n+1)P_{n}(\cos\theta )=0,$$

and we can conclude from Eq. (7) that

$$\Delta_{M}P_{n}(\cos\theta )=-n(n+1)P_{n}(\cos\theta )$$

on the surface of the sphere. An illustrative example of $P_{6}(\cos\;\theta )$ on the sphere is shown in Fig. 3(a).

### 3.1 Helmholtz equation

To illustrate that the discretisation retains the spatial convergence properties of the spectral/*hp* element method we extend the result above to solve the Helmholtz equation,

(8) $$\Delta_{M}u(x)-\lambda u(x)=f(x),\;x\in \Omega ,$$

$$u(x)=g_{D}(x),\;x\in \partial \Omega ,$$

on the spherical patch $\Omega =\{S^{2}(r,\theta ,\phi )|(\theta ,\phi )\in [-\frac{\pi }{4},\frac{\pi }{4}]\times [-\frac{\pi }{2},\frac{\pi }{2}],\;r=1\}$. Extending our analysis for the Laplace equation above, we choose the forcing function

$$f=-\left(\lambda +n(n+1)\right)P_{n}(\cos\theta )$$

and impose Dirichlet boundary conditions

$$g_{D}(x)=P_{n}(\cos\theta ),$$

which, by construction, has an exact solution on *Ω* of

$$u(x)=P_{n}(\cos\theta ).$$

The computational meshes used for this example consist of either 36 triangular elements, as illustrated in Fig. 3(b), or 18 quadrilateral elements. On each element polynomial bases with maximum degrees of P=1 through P=8 are considered. Both the triangular and quadrilateral discretisations result in the same number of degrees of freedom for a given *P*, since elemental edge modes are coupled under the continuous Galerkin formulation. In a curvilinear manifold, the accuracy of a numerical solution is dependent on both the ability of the expansion basis to capture the solution and on the geometric accuracy of parametric coordinate mappings and consequently the differential operators. The coordinate mappings $\chi_{i}$, from the two-dimensional reference regions to the physical elements are constructed in terms of high-order bases much like those used to represent the solution. The derivatives of these mappings and the entries of the Jacobian matrix are then evaluated pointwise at the quadrature points used for integration in the solution space when constructing the differential operators. The quality of the resulting solution is therefore a function of the characteristic mesh element size (*h*), the solution polynomial order (*P*) and the order of the geometric representation ($P_{g}$) of the elements.

**Fig. 4:**
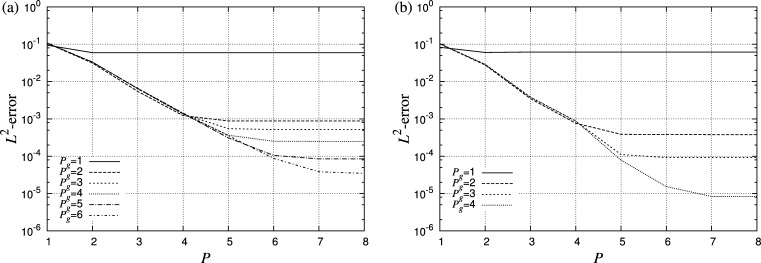
Error of the numerical solution in the $L^{2}$ norm with polynomial order for the Helmholtz problem given by Eq. (8) on a patch of the unit sphere, shown in Fig. 3(b), using either (a) curvilinear triangular elements, or (b) curvilinear quadrilateral elements. Exponential convergence is obtained with increasing polynomial order *P* on both meshes across a range of geometric orders $P_{g}$. Errors saturate when the geometric error dominates the spatial discretisation error.

**Fig. 5:**
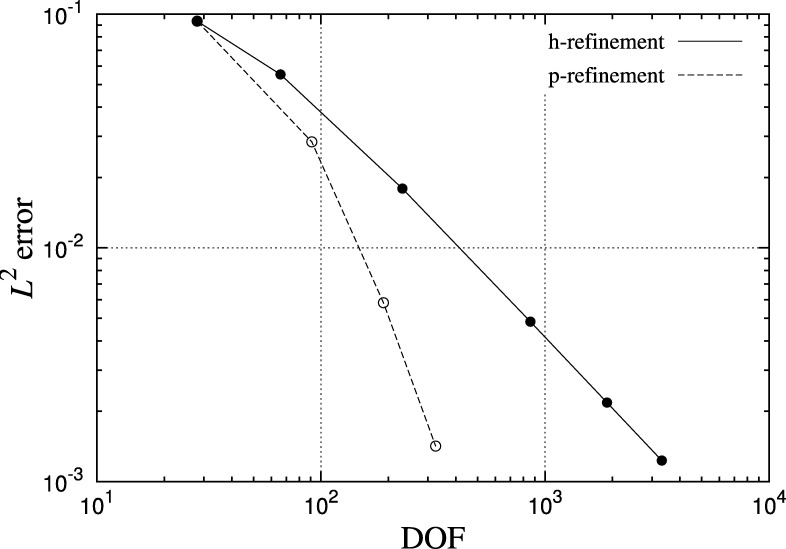
Comparison of solution convergence for the Helmholtz problem using triangular meshes. $L_{2}$-error is plotted against number of degrees of freedom on a log-log scale for the 18 element mesh shown in Fig. 3(b).

Fig. 4 shows the error in the solution to the Helmholtz problem given in Eq. (8) on a number of triangular and quadrilateral meshes, ranging in order from $P_{g}=1$ to $P_{g}=6$. A fixed number of curvilinear elements are used across the range of $P_{g}$ for each shape. Errors are computed as the difference between the computed solution at the mesh points and the exact solution at the corresponding radially projected point on the true surface. An exponential reduction in error is observed for all cases, in line with the spectral convergence properties expected of the method. The error is seen to saturate when the error introduced by the representation of the geometry dominates the error from the spectral/*hp* element discretisation of the solution. In this example planar elements capture the geometry very poorly and introduce significant errors in the differential operators, while higher values of $P_{g}$ resolve the geometry much better and significantly reduce the error. Quadrilateral tessellations attain a lower error than their triangular counterparts in general. The convergence of the solution using both mesh and polynomial refinement is shown in Fig. 5. In the case of mesh refinement, both the geometry and solution expansions are linear. *P*-refinement obtains more rapid convergence than *h*-refinement.

### 3.2 Parabolic problem

The second example is a time-dependent diffusion problem on $S^{2}$. The mesh consists of 1456 quadrilateral elements with $P_{g}=5$ and the solution is represented using polynomial expansions up to order P=5 on each element. We extend our solution of the Laplace equation to solve the heat equation and define our initial value problem to be

(9) $$\frac{\partial u}{\partial t}(x,t)=\varepsilon \Delta_{M}u(x,t),\;x\in \Omega ,$$

(10) $$u(x,0)=P_{n}(\cos\theta ).$$

**Fig. 6:**
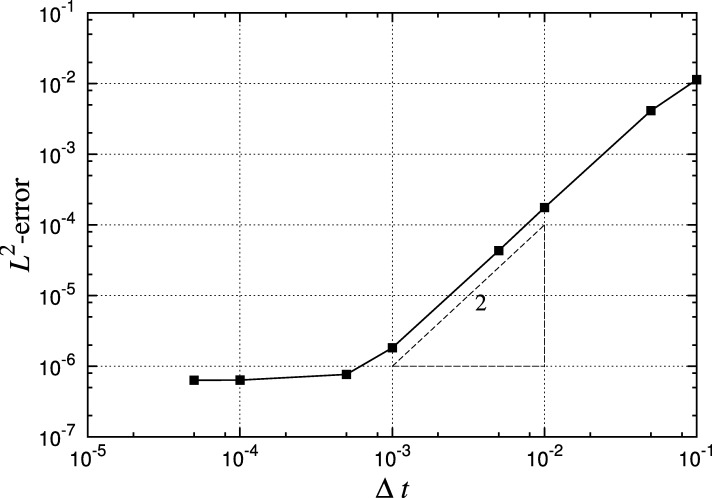
Error in solving diffusion equation for *T*=0.1 time units, showing time convergence of a second-order backwards difference formula implicit scheme, *P*=5, $P_{g}=5$ and *ε*=0.1, with an initial condition of $u_{0}=P_{6}(\cos\;\theta )$, on a 1452-quadrilateral spherical mesh.

This has an analytic solution of the form $u(x,t)=P_{n}(\cos\;\theta )e^{-n(n+1)\varepsilon t}$. A representative illustration of this function on the spherical domain can be seen in Fig. 3(a). Fig. 6 shows the convergence of the solution in time using the second-order implicit backwards difference formula. The correct second-order convergence is obtained until the spatial discretisation and geometric errors begin to dominate.

## 4 Application to cardiac electrophysiology

The most prevalent PDE model used to describe electrical propagation in the heart is the monodomain reaction–diffusion equation. The reaction term is a system of Ordinary Differential Equations (ODEs) which characterise the flow of ions in and out of individual cells. The diffusion component of the system describes the propagation of the electrochemical action potential between cells in the tissue. Since the myocardium is fibrous in nature, this diffusion is highly anisotropic and can lead to conduction velocities which are an order of magnitude higher in the direction of the fibre compared to the transverse direction in some types of cardiac tissue. The PDE is defined as

$$\beta \left(C_{m}\frac{\partial u}{\partial t}+J\right)=\nabla \cdot (\sigma \nabla u),$$

$$\frac{\partial v}{\partial t}=f(u,v),$$

where u(x,t) is the potential difference across the membrane of a cell, v(x,t) is the cell model state, *β* is the cellular surface-to-volume ratio, $C_{m}$ is the membrane capacitance and $J(x,t)=J_{\mathrm{ion}}(x,t)+J_{s}(x,t)$ is the total outward flowing current from a cell as given by the cell model f(u,v), and the stimulus current used to activate the system.

The diffusivity tensor ***σ*** reflects the coupling between adjacent cells through gap junctions and the orientation of cells within the tissue. This directly affects the propagation velocity of wavefronts with greater coupling leading to higher conduction velocities. We consider two choices for the diffusivity tensor: the isotropic homogeneous case where there are no spatial variations in conductivity and fibre direction is not accounted for, and an anisotropic heterogeneous case where this information is included in the model.

Cardiac cells maintain a resting cellular transmembrane potential of approximately −85 mV, but the movement of ions leads to a change in the potential difference across the membrane of the cell and the development of an action potential, which numerically is described by the system of ODEs. A broad choice of ionic and phenomenological cell models exist which capture the characteristics of different types of cardiac cells with differing levels of biophysical accuracy. The model chosen for the atrium simulations presented here is the Courtemanche et al. model [16] consisting of 20 ODEs and derived from experimental recordings on human and animal atrial cells.

The particular numerical challenges which arise when modelling cardiac electrophysiology are: the stiffness of the cell model and the necessary time-step restrictions; the resulting steep spatial gradients which need to be effectively captured by the spatial discretisation in order to correctly predict conduction velocities; and the geometric complexity of accurately representing the anatomy. As a consequence, the computational cost of such whole-chamber models is very high, often requiring extended time on large clusters.

The computational mesh *Ω*, used for the simulations presented here, is obtained through segmentation of magnetic resonance angiography (MRA) images. A surface mesh is then generated, which is post-processed to ensure appropriate element density and uniformity to capture the sharp gradient in the wavefronts using high-order elements. The pulmonary veins are initially closed over in the generated surface mesh so these are opened to correctly model human physiology. No-flux Neumann boundary conditions are imposed on the edge of these pulmonary vein sleeves. The other main feature is the appendage, a finger-like extension from the main body of the atrium. Each element is augmented with high-order geometric information using the spherigon technique [30] which, in the limit $P_{g}\to \infty$, produces a $C^{1}$-smooth surface. In this method, vertex normals are calculated as the average of the surrounding face normals and these are then fitted to a sphere to define the curvature of each element.

**Fig. 7:**
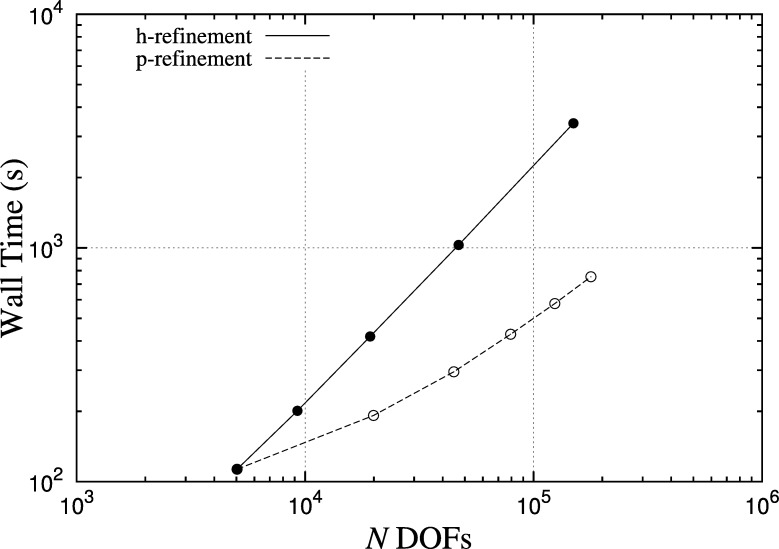
Comparison of solver scaling for *h*- and *p*-refinement based on values from Table 1. Timings are for 600 ms simulations, Δ*t*=0.02, of the isotropic case on 80 cores of an SGI Altix ICE 8200 EX. Initial discretisation with smallest number of DOFs is *h*=1.4, *P*=1.

The spatial and temporal discretisation is controlled by a number of parameters. A convergence study provides guidance on suitable choices of these parameters for achieving accurate solutions efficiently. Results of these tests are shown in Table 1 where for each test we report the time at which complete activation of the atrium occurs, as well as the time of the simulation, on 80 cores of an SGI Altix ICE 8200 EX. Activation time is used as a metric for accuracy, since conduction velocity is particularly sensitive to spatial resolution. For each mesh, the original STL description of the atrial surface was remeshed using Gmsh using elements of the specified characteristic length *h*. Based on this data, a suitable choice of parameters would be h=1.4 mm, P=5, Δt=0.02 which achieves an accuracy of within 5%. We also plot in Fig. 7 a comparison of the performance scaling of the solver when using *h*- and *p*-refinement using a subset of the data in Table 1. Increasing resolution by refining the polynomial order results in faster simulations for comparable degrees of freedom and wall time increases at a slower rate relative to using *h*-refinement. We note that although these data include timings of the whole solve (linear system and cell model evaluation), the ionic model is evaluated pointwise and therefore it can be reasonably assumed that its computational cost scales linearly with the number of degrees of freedom and by the same factor in both cases.

### 4.1 Isotropic propagation

**Fig. 8:**
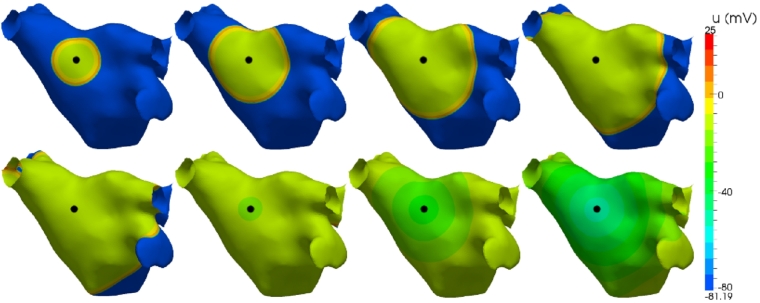
Electrical propagation across an atrium from a circular stimulus in the left-superior wall (black dot). Images show transmembrane voltage at 20 ms, 40 ms, 60 ms and 80 ms (top row), and 100 ms, 150 ms, 200 ms and 250 ms (bottom row) after initial stimulus. (For interpretation of the references to colour in this figure, the reader is referred to the web version of this article.)

In the isotropic case, the conductivity of the tissue is fixed at σ(x)=Iσ for a physiologically appropriate choice of the scalar $\sigma =0.13341\;\text{mS}\;{\text{mm}}^{-1}$. Fig. 8 shows a sequence of snapshots characterising the depolarisation propagation through the atrium. The coloured contours indicate the value of the transmembrane potential *u* ranging from a depolarised +25 mV (red) down to a polarised −81 mV (blue). Initial activation is induced through a 2 ms stimulus current $J_{s}$, of strength 50 μA/mm^2^ applied to a region $\Omega_{s}=\Omega \cap S^{2}(x_{s},r_{s})$, for some position $x_{s}$ and radius $r_{s}$ of stimulation, as indicated by the black dot in the figure. Activation is characterised by rapid depolarisation followed by a gradual recovery of the polarised state. The activation wavefront propagates uniformly across the surface from the region of activation, following the contours of the surface. Conduction velocity is uniformly 0.5 m/s and complete activation of the atrium occurs after 182 ms. The wavelength of the depolarisation wave is typically greater than the diameter of the atrium in a healthy heart, inherently reducing the opportunity for reentry and arrhythmias. Atrial tissue in a depolarised plateau phase after the wavefront passes cannot support further activation until it has repolarised.

### 4.2 Anisotropic heterogeneous propagation

**Fig. 9:**
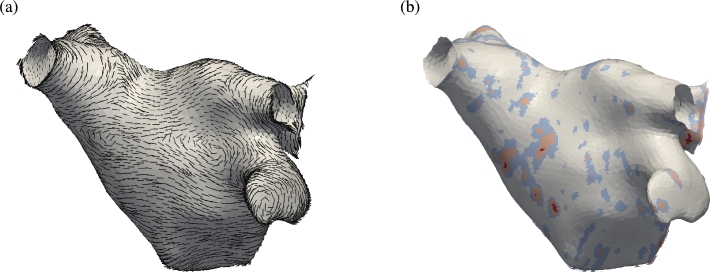
(a) Representative fibre orientation of the human left atrium used to prescribe anisotropic conductivities in the model. (b) Conductivity map (***σ***) derived from late gadolinium DE-MRA imaging intensities. (For interpretation of the references to colour in this figure, the reader is referred to the web version of this article.)

**Fig. 10:**
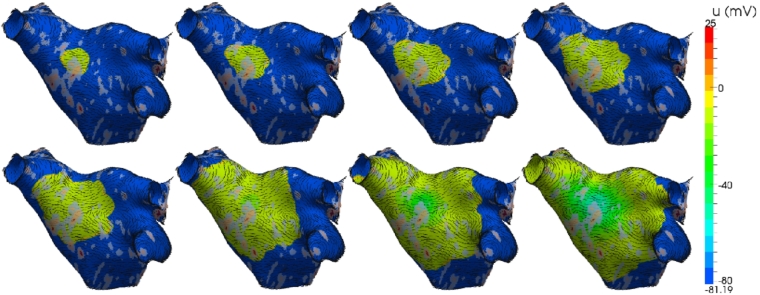
Electrical propagation across the atrium, incorporating scar information and fibre orientation, from a circular stimulus in the left-superior wall. Images show transmembrane voltage at 20 ms, 40 ms, 60 ms and 80 ms (top row), and 100 ms, 150 ms, 200 ms and 250 ms (bottom row) after the initial stimulus.

The isotropic model is now extended with information describing scarring of the myocardium and fibre orientation. This data represents electrophysiological characteristics of the tissue and so better reflects the true activation pattern of the atrium. Fibre orientation is prescribed using histological examination of ex-vivo human atria and is shown in Fig. 9(a). At each quadrature point the diffusion tensor $\sigma_{ii}$ is defined as the *i*-th component of the unit vector in the primary fibre direction. Fibre direction is oriented around the pulmonary veins and includes the main Bachmann fibre bundle which is the primary connection to the right atrium and runs laterally relative to the perspective of the figure and through the point of stimulus. Conductivities are set to reflect physiologically measured values from the literature, with an anisotropic ratio of 1:8 and preferential conductivity along the fibres. Scar tissue can be obtained through late-gadolinium enhanced magnetic resonance imaging (LG-MRI) where higher intensity voxels correlate with the location of scarred tissue. Healthy tissue is defined as intensities equal to, or less than, the mean blood pool intensity. Full scar is defined as intensities of 3 standard deviations (S.D.) above the blood pool mean intensity. Representative scar data is shown in Fig. 9(b) where grey indicates low intensity and red indicates intensities of 3 S.D. above the blood pool mean. Electrical propagation in an atrium incorporating scar tissue and fibre orientation is shown in Fig. 10 with the same stimulus protocol applied as for the isotropic case in Fig. 8. Activation wavefronts now propagate in a non-uniform manner and advance faster along the direction of fibres. Conduction is slowed by partial scar and propagates around full scar. Average conduction velocities are significantly reduced and increase the time taken to fully activate the atrium.

## 5 Discussion

In this paper we have outlined the construction of a high-order finite element method on arbitrary smooth codimension-1 surfaces embedded in a three-dimensional space. We use a geometric tensor approach to give a rigorous definition (see Appendix A) and compare it with an extension of the conventional Euclidean construction of planar geometric terms in finite element methods. We confirm the validity of the numerical and geometric approximations through example test cases. Finally, the method is demonstrated using a more complex biophysical modelling problem in cardiac electrophysiology.

The test cases confirm that the high-order discretisation retains exponential convergence properties with increasing geometric and expansion polynomial order if both the solution and true surface are smooth. Errors are found to saturate when the geometric errors, due to the parametrisation of the surface elements, begin to dominate the temporal and spatial discretisation errors. For the smooth solutions considered as test cases, the results show that this dominance of geometric errors quickly limits the effectiveness of further increases in the number of degrees of freedom, either through mesh refinement or higher solution polynomial orders. Increasing the order of the geometry parametrisation reduces the geometric error. The analytic test cases presented here use a coarse curvilinear mesh; for applications, meshes are typically more refined in order to capture features in the solution and so will better capture the geometry and consequently reduce this lower bound on the solution error. If the solution is not smooth, we do not expect to see rapid convergence. In the case that the solution is smooth, but the true surface is not, then exponential convergence with *P* and $P_{g}$ can only be achieved if, and only if, the discontinuities are aligned with element boundaries. However, if discontinuities lie within an element, convergence will be limited by the geometric approximation, since the true surface cannot be captured. In the cardiac problem, we consider both the true surface and solution to be smooth.

Numerical convergence tests on the atrium reflect the significant speed-up obtained using high-order discretisations in comparison to linear discretisations for the same level of accuracy. While a proportion of this speed-up can be attributed to the faster convergence rate of polynomial refinement over mesh refinement, high-order methods are significantly faster for comparable numbers of degrees of freedom. This is most likely due to the increased data locality of elemental regions and therefore greater CPU cache coherence when performing operations element-wise, compared to the cost of memory indirection when evaluating a large sparse matrix in the linear case.

The general metric formulation was compared with an extension of a traditional two-dimensional finite element method implementation to the embedded manifold. While the two approaches can be seen to be mathematically equivalent, the metric formulation possesses greater implementational simplicity. The metric tensor is of size 2×2 compared to the 2×3 matrix of factors resulting from extending the conventional formulation. For a large mesh, this may be a significant storage consideration. Furthermore, in the general case when the geometric definition and the quadrature points do not share the same points distribution, interpolation is necessary on each usage of the Jacobian or metric factors. Consequently, the proposed metric tensor approach will have a reduced computational cost.

## Figures and Tables

**Table 1:** Results of a convergence study for a human atrium mesh with isotropic conductivity for a range of values of polynomial order *P*, element characteristic length *h* and time-step Δ*t*. Timings show absolute wall time for a 600 ms simulation on 80 cores of an SGI Altix ICE 8200 EX.

*h* (mm)	*P*	$N_{\mathrm{elmt}}$	DOF	Δ*t* (ms)	$A_{\max}$ (ms)	*T*
0.6	1	297 898	149 493	0.02	172	56 m 56 s
0.8	1	93 309	46 960	0.02	194	17 m 10 s
1.0	1	38 144	19 268	0.02	241	6 m 58 s
1.2	1	18 222	9246	0.02	321	3 m 21 s
1.4	1	9869	5033	0.02	442	1 m 53 s

0.8	2	93 309	187 232	0.02	175	29 m 55 s
1.0	2	38 144	76 684	0.02	186	12 m 16 s
1.2	2	18 222	36 717	0.02	211	5 m 55 s
1.4	2	9869	19 938	0.02	250	3 m 12 s

0.8	3	93 309	420 811	0.02	176	45 m 58 s
1.0	3	38 144	172 245	0.02	178	18 m 25 s
1.2	3	18 222	82 410	0.02	187	8 m 48 s
1.4	3	9869	44 712	0.02	207	4 m 55 s

1.4	4	9869	79 355	0.02	188	7 m 07 s

1.4	5	9869	123 867	0.10	193	1 m 57 s
1.4	5	9869	123 867	0.08	190	2 m 26 s
1.4	5	9869	123 867	0.06	187	3 m 14 s
1.4	5	9869	123 867	0.04	185	4 m 48 s
1.4	5	9869	123 867	0.02	182	9 m 38 s
1.4	5	9869	123 867	0.01	180	18 m 58 s
1.4	5	9869	123 867	0.005	177	39 m 21 s

1.4	6	9869	178 248	0.02	178	12 m 33 s

## Appendix A Differential geometry formulation of the Laplace operator

We outline a rigorous derivation of the Laplace–Beltrami operator. Further to the notation used in Section 2, we use the convention that indices appearing once in the upper position and once in the lower position are considered dummy indices and are implicitly summed over their range, while non-repeated indices are considered free to take any value. Derivatives are also denoted using the lower-index comma notation, for example $g_{ij,k}$. With this in mind, we now construct the fundamental differential operators we require for a 2-dimensional manifold embedded in a 3-dimensional space. In order to express these operators in curvilinear coordinates we start by assuming that we have a smooth surface parametrisation given by

$$x\left(\xi^{1},\xi^{2}\right):=\left(x^{1}\left(\xi^{1},\xi^{2}\right),x^{2}\left(\xi^{1},\xi^{2}\right),x^{3}\left(\xi^{1},\xi^{2}\right)\right).$$

Next we will define the Jacobian of ***x*** as the tensor

$$J_{i}^{j}=\frac{\partial x^{j}}{\partial \xi^{i}}$$

where $J_{i}^{j}$ can be viewed as a covariant surface vector (by fixing the upper index) or as a contravariant space vector (by fixing the lower index). The surface metric tensor $g_{ij}$ can be defined in terms of the $J_{i}^{j}$ as

(A.1) $$g_{ij}=\sum_{k=1}^{3}J_{i}^{k}J_{j}^{k},$$

which can be considered to transform a contravariant quantity to a covariant quantity. Similarly the conjugate tensor $g^{ij}$, which does the reverse transformation, is given by

(A.2) $$g^{11}=g_{22}/g,\;g^{12}=g^{21}=-g_{12}/g,\;g^{22}=g_{11}/g,$$

where *g* is the determinant of $g_{ij}$. The metric tensor and its conjugate satisfy the condition

$$\delta_{i}^{j}=g_{ik}g^{jk}=\left\{ \begin{array}{cc} 1, & \text{if }i=j,\\ 0, & \text{if }i\neq j. \end{array}\right.$$

To construct the divergence operator we will also need the derivative of *g* with respect to components of the metric, $g_{ij}$. We know that ***g*** is invertible and from linear algebra we have that the inverse of the metric (A.2) satisfies $g^{-1}=\frac{1}{g}{\tilde{g}}^{⊤}$, where $\tilde{g}$ is the cofactor matrix of ***g***. Therefore ${\tilde{g}}^{⊤}=gg^{-1}$, or in components ${\tilde{g}}^{ij}=g{\left(g^{-1}\right)}_{ji}$. Using Jacobiʼs formula for the derivative of a matrix determinant with respect to its entries, and since ***g*** is invertible, the derivative of the metric determinant is

(A.3) $$\frac{\partial g}{\partial g_{ij}}=\mathrm{tr}\left({\tilde{g}}^{⊤}\frac{\partial g}{\partial g_{ij}}\right)={\tilde{g}}^{ij}=g{\left(g^{-1}\right)}_{ji}=gg^{ij}.$$

### A.1 Divergence operator

The partial derivative of a tensor with respect to a manifold coordinate system is itself not a tensor. In order to obtain a tensor, one has to use the *covariant* derivative, defined below. The covariant derivative of a contravariant vector is given by

(A.4) $$\nabla_{k}a^{i}=a_{,k}^{i}+a^{j}\Gamma_{jk}^{i},$$

where $\Gamma_{jk}^{i}$ are *Christoffel symbols of the second kind*. The *Christoffel symbols of the first kind* are defined by

$$\Gamma_{ijk}=\frac{1}{2}[g_{kj,i}+g_{ik,j}-g_{ij,k}].$$

Here we note that $\Gamma_{ijk}$ is symmetric in the first two indices. To obtain the Christoffel symbols of the second kind we formally raise the last index using the conjugate tensor,

(A.5) $$\Gamma_{ij}^{l}=\Gamma_{ijk}g^{kl}$$

which retains the symmetry in the lower two indices. We can now express the derivatives of the metric tensor in terms of the Christoffel symbols as

$$g_{ij,k}=\Gamma_{ikj}+\Gamma_{jki}=g_{lj}\Gamma_{ik}^{l}+g_{li}\Gamma_{jk}^{l}.$$

We now define the divergence operator on the manifold, $\nabla \cdot X=\nabla_{k}X^{k}$. Consider first the derivative of the determinant of the metric tensor *g* with respect to the components of some local coordinates system $\xi^{1},\xi^{2}$. We apply the chain rule, making use of the derivative of the metric tensor determinant with respect to components of the metric (A.3) and the relationship (A.5), to get

$$\frac{\partial g}{\partial \xi^{k}}=\frac{\partial g}{\partial g_{ij}}\frac{\partial g_{ij}}{\partial \xi^{k}}=gg^{ij}g_{ij,k}=gg^{ij}(\Gamma_{ikj}+\Gamma_{jki})=g\left(\Gamma_{ik}^{i}+\Gamma_{jk}^{j}\right)=2g\Gamma_{ik}^{i}.$$

We can therefore express the Christoffel symbol $\Gamma_{ik}^{i}$ in terms of this derivative as

(A.6) $$\Gamma_{ik}^{i}=\frac{1}{2g}\frac{\partial g}{\partial \xi^{k}}=\frac{1}{\sqrt{g}}\frac{\partial \sqrt{g}}{\partial \xi^{k}}.$$

Finally, by substituting for $\Gamma_{ik}^{i}$ in the expression for the divergence operator

$$\nabla_{k}X^{k}=X_{,k}^{k}+X^{i}\Gamma_{ki}^{k}=X_{,k}^{k}+X^{k}\Gamma_{ik}^{i}=X_{,k}^{k}+X^{k}\frac{1}{\sqrt{g}}{\left(\sqrt{g}\;\right)}_{,k}$$

and we can deduce a formula for divergence of a contravariant vector as

(A.7) $$\nabla \cdot X=\nabla_{k}X^{k}=\frac{{\left(X^{k}\sqrt{g}\;\right)}_{,k}}{\sqrt{g}}.$$

### A.2 Laplacian operator

The covariant derivative (gradient) of a scalar on the manifold is identical to the partial derivative, $\nabla_{k}\phi =\phi_{,k}$. To derive the Laplacian operator we need the contravariant form of the covariant gradient above which can be found by raising the index using the metric tensor, giving

(A.8) $$\nabla^{k}\phi =g^{kj}\phi_{,j},$$

and substituting (A.8) for $X^{k}$ in (A.7) to get the Laplacian operator on the manifold as

(A.9) $$\Delta_{M}\phi =\frac{1}{\sqrt{g}}{\left(\sqrt{g}g^{ij}\phi_{,j}\right)}_{,i}.$$

### A.3 Anisotropic Laplacian operator

Anisotropic diffusion is important in many applications. In the ambient Euclidean space, this can be represented by a diffusivity tensor ***σ*** in the Laplacian operator as

$$\Delta_{M}=\nabla \cdot \sigma \nabla .$$

On our manifold, we seek the generalisation of (A.9), in the form

$${\tilde{\Delta }}_{M}\phi =\nabla_{j}{\tilde{\sigma }}_{i}^{j}\nabla^{i}\phi ,$$

where the ${\tilde{\sigma }}_{i}^{j}$ are entries in the surface diffusivity. For a contravariant surface vector $a^{j}$ we can find the associated space vector $A^{i}$ as $A^{i}=J_{j}^{i}a^{j}$. Similarly if $A_{i}$ is a covariant space vector, then $a_{j}=J_{j}^{i}A_{i}$ is a covariant surface vector. Using these we can construct the anisotropic diffusivity tensor $\tilde{\sigma }$ on the manifold by constraining the ambient diffusivity tensor ***σ*** to the surface. The contravariant surface gradient $\nabla^{i}\phi$ is mapped to the corresponding space vector, which lies in the tangent plane to the surface. This is scaled by the ambient diffusivity and then projected back to a covariant surface vector. Finally, we use the conjugate metric to convert back to a contravariant form. The resulting surface Laplacian is

$${\tilde{\Delta }}_{M}\phi =\nabla_{m}g^{lm}J_{l}^{k}\sigma_{jk}J_{i}^{j}\nabla^{i}\phi .$$

Following on from this we deduce that

$${\tilde{\sigma }}_{i}^{j}=g^{jm}J_{m}^{l}\sigma_{lk}J_{i}^{k}.$$

It can be seen that in the case of isotropic diffusion that ${\tilde{\sigma }}_{i}^{j}=\delta_{j}^{i}\Leftrightarrow \sigma =I$,

$${\tilde{\sigma }}_{i}^{j}=g^{im}J_{m}^{k}\sigma_{lk}J_{j}^{l}=g^{im}J_{m}^{k}J_{j}^{k}=g^{im}g_{mj}=\delta_{j}^{i}.$$

## References

[1] Stam J. (2003). Flows on surfaces of arbitrary topology. ACM Trans. Graph..

[2] Sbalzarini I., Hayer A., Helenius A., Koumoutsakos P. (2006). Simulations of (an) isotropic diffusion on curved biological surfaces. Biophys. J..

[3] Burger M. (2009). Finite element approximation of elliptic partial differential equations on implicit surfaces. Comput. Vis. Sci..

[4] Dziuk G. (1988). Finite elements for the Beltrami operator on arbitrary surfaces. Partial Differential Equations and Calculus of Variations.

[5] Holst M. (2001). Adaptive numerical treatment of elliptic systems on manifolds. Adv. Comput. Math..

[6] Dziuk G., Elliott C.M. (2007). Surface finite elements for parabolic equations. J. Comput. Math..

[7] Calhoun D., Helzel C. (2009). A finite volume method for solving parabolic equations on logically Cartesian curved surface meshes. SIAM J. Sci. Comput..

[8] Osher S., Fedkiw R. (2002). Level Set Methods and Dynamic Implicit Surfaces.

[9] Macdonald C., Ruuth S. (2008). Level set equations on surfaces via the closest point method. J. Sci. Comput..

[10] Macdonald C., Ruuth S. (2009). The implicit closest point method for the numerical solution of partial differential equations on surfaces. SIAM J. Sci. Comput..

[11] Greer J., Bertozzi A., Sapiro G. (2006). Fourth order partial differential equations on general geometries. J. Comput. Phys..

[12] Ruuth S., Merriman B. (2008). A simple embedding method for solving partial differential equations on surfaces. J. Comput. Phys..

[13] Dziuk G., Elliott C.M. (2008). Eulerian finite element method for parabolic PDEs on implicit surfaces. Interfaces Free Bound..

[14] Deckelnick K., Dziuk G., Elliott C.M., Heine C.J. (2010). An h-narrow band finite-element method for elliptic equations on implicit surfaces. IMA J. Numer. Anal..

[15] Greer J. (2006). An improvement of a recent Eulerian method for solving PDEs on general geometries. J. Sci. Comput..

[16] Courtemanche M., Ramirez R., Nattel S. (1998). Ionic mechanisms underlying human atrial action potential properties: insights from a mathematical model. Am. J. Physiol., Heart Circ. Physiol..

[17] Giraldo F. (2001). A spectral element shallow water model on spherical geodesic grids.

[18] Taylor M., Tribbia J., Iskandarani M. (1997). The spectral element method for the shallow water equations on the sphere. J. Comput. Phys..

[19] Diewald U., Preußer T., Rumpf M. (2000). Anisotropic diffusion in vector field visualization on euclidean domains and surfaces. IEEE Trans. Vis. Comput. Graph..

[20] Arthurs C.J., Bishop M.J., Kay D. (2012). Efficient simulation of cardiac electrical propagation using high order finite elements. J. Comput. Phys..

[21] Aris R. (1989). Vectors, Tensors, and the Basic Equations of Fluid Mechanics.

[22] Karniadakis G., Sherwin S. (2005). Spectral/hp Element Methods for CFD.

[23] Dubiner M. (1991). Spectral methods on triangles and other domains. J. Sci. Comput..

[24] Sherwin S.J., Karniadakis G.E. (1996). Tetrahedral *hp* finite elements: Algorithms and flow simulations. J. Comput. Phys..

[25] (2012). Nektar++. http://www.nektar.info.

[26] Vos P.E., Sherwin S.J., Kirby R.M. (2010). From *h* to *p* efficiently: Implementing finite and spectral/*hp* element methods to achieve optimal performance for low- and high-order discretisations. J. Comput. Phys..

[27] Fischer P., Lottes J., Pointer D., Siegel A. (2008). Petascale algorithms for reactor hydrodynamics. J. Phys. Conf. Ser..

[28] Ascher U.M., Ruuth S.J., Wetton B.T. (1995). Implicit–explicit methods for time-dependent partial differential equations. SIAM J. Numer. Anal..

[29] Vos P.E., Eskilsson C., Bolis A., Chun S., Kirby R.M., Sherwin S.J. (2011). A generic framework for time-stepping partial differential equations (PDEs): general linear methods, object-oriented implementation and application to fluid problems. Int. J. Comput. Fluid Dyn..

[30] Volino P., Thalmann N. (1998). The spherigon: a simple polygon patch for smoothing quickly your polygonal meshes. Computer Animation 98, Proceedings.

